# Case report: endocarditis or catheter-related right atrial thrombosis in a patient undergoing chemotherapy for Hodgkin lymphoma: diagnostic challenges and therapeutic dilemmas

**DOI:** 10.1093/ehjcr/ytaf570

**Published:** 2025-11-01

**Authors:** Sokpich Ren, Minh-Tam Bailly, Emmanuelle Berthelot, Fabrice Bauer, Safwane El hatimi

**Affiliations:** Kremlin-Bicêtre University Hospital, Assistance Publique-Hôpitaux de Paris, 78 Rue du Général Leclerc, Le Kremlin-Bicêtre, Paris 94270, France; Kremlin-Bicêtre University Hospital, Assistance Publique-Hôpitaux de Paris, 78 Rue du Général Leclerc, Le Kremlin-Bicêtre, Paris 94270, France; Kremlin-Bicêtre University Hospital, Assistance Publique-Hôpitaux de Paris, 78 Rue du Général Leclerc, Le Kremlin-Bicêtre, Paris 94270, France; Kremlin-Bicêtre University Hospital, Assistance Publique-Hôpitaux de Paris, 78 Rue du Général Leclerc, Le Kremlin-Bicêtre, Paris 94270, France; Kremlin-Bicêtre University Hospital, Assistance Publique-Hôpitaux de Paris, 78 Rue du Général Leclerc, Le Kremlin-Bicêtre, Paris 94270, France

**Keywords:** Infective endocarditis, Catheter-related right atrial thrombosis (CRAT), Cancer, Chemotherapy, Hodgkin lymphoma, Port-a-cath, Case report

## Abstract

**Background:**

Infective endocarditis (IE) in oncology patients represents a clinical challenge due to overlapping risk factors, diagnostic complexities, and treatment dilemmas, particularly in patients undergoing chemotherapy. We present a case that underscores the diagnostic difficulties distinguishing between IE and catheter-related right atrial thrombosis, emphasizing decision-making complexities regarding chemotherapy interruption.

**Case summary:**

A 29-year-old female with known Stage III Ab Hodgkin lymphoma, previously treated with chemotherapy and recent reimplantation of a port-a-cath presented with a 1-week history of fever, was diagnosed with febrile aplasia and treated with broad-spectrum antibiotic therapy. Initial two sets of blood cultures were negative. Transthoracic echocardiography demonstrated a mobile mass attached to the lateral tricuspid annulus within the right atrium. Thoraco-abdominopelvic computed tomography revealed distal pulmonary embolism. After 2 weeks of antibiotic and curative anticoagulant, complete resolution of intracardiac mass made the diagnosis of CRAT more likely and multidisciplinary team decided on discontinuation of antibiotic, continuation of anticoagulant, and resumption of chemotherapy, which was suspended for 2 weeks following suspicion of IE.

**Discussion:**

In the initial management of an intracardiac mass in the context of scheduled chemotherapy, two primary diagnostic hypotheses arise: infectious endocarditis and intracardiac thrombus. Ultimately, interdisciplinary consultation with cardiovascular infectious disease experts suggested a thrombotic lesion associated with abnormal jet flow supported by the absence of clinical and biological signs of infection. Prompt discontinuation of antibiotics, initiation of appropriate anticoagulation therapy, and close monitoring enabled safe resumption of chemotherapy.

Learning points

- Importance of early multidisciplinary collaboration on suspected infective endocarditis (IE) in cancer patients
- Diagnostic complexity differentiating catheter-related atrial thrombosis from IE in patients with intracardiac catheters
- Balancing risks of chemotherapy delay against potential infectious exacerbation in cancer patients

## Introduction

Central venous catheters (CVCs), including port-a-caths, are widely used in oncology but can lead to serious complications such as catheter-related right atrial thrombosis (CRAT) and infective endocarditis (IE), which share overlapping features and pose diagnostic and therapeutic challenges.

Infective endocarditis is more frequent in cancer patients, with atypical features due to immunosuppression, nosocomial infections, and invasive procedures, often delaying diagnosis. A European registry showed worse outcomes, higher embolic risk, and mortality compared to non-cancer patients.^1^

Catheter-related right atrial thrombosis is a known complication of long-term CVCs, especially when the tip lies in the atrium, promoting thrombus via endothelial trauma and flow disturbances (‘jet lesion’).^2^ Catheter-related right atrial thrombosis can be asymptomatic or present as PE or superior vena cava (SVC) syndrome, and may mimic IE when cultures are negative.

Due to overlapping features between CRAT and IE in immunocompromised cancer patients, accurate diagnosis is crucial to guide treatment—anticoagulation, antibiotics, device removal, or chemotherapy suspension. We present a case illustrating these challenges in a young woman treated for Hodgkin lymphoma.

## Summary figure

**Figure:**
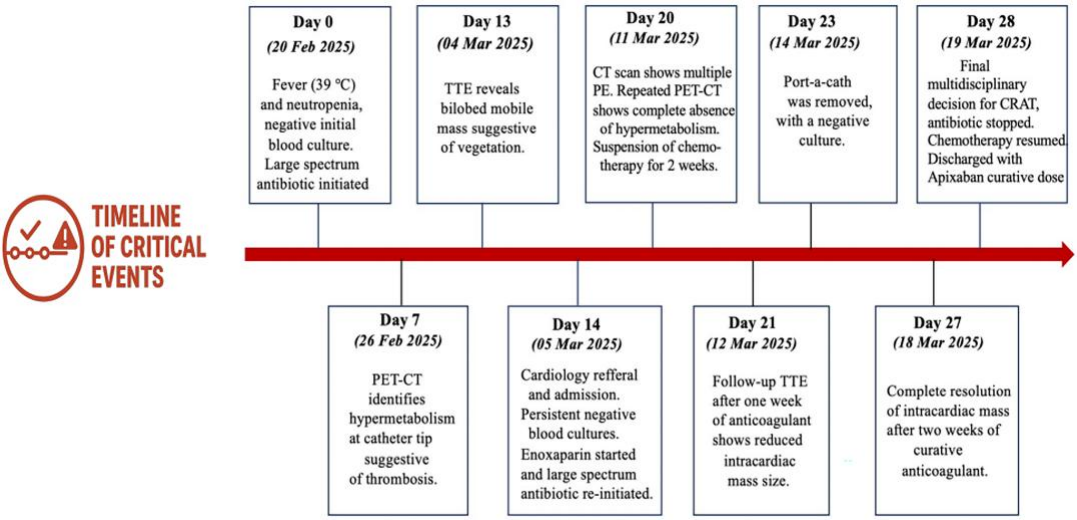
Timeline of critical clinical events. Sequential summary of key diagnostic and therapeutic milestones in a 29-year-old patient undergoing chemotherapy. Initial presentation with fever and neutropenia (Day 0) led to broad-spectrum antibiotics. Positron emission tomography–computed tomography on Day 7 revealed hypermetabolism at the catheter tip, suggesting infection. A bilobed intracardiac mass was later visualized on Transthoracic echocardiography (TTE) (Day 13), initially suggestive of endocarditis. Despite negative cultures (Day 14), antibiotic therapy was continued, suspecting thrombosis enoxaprine was started. CT (Day 20) showed multiple pulmonary emboli and absence of metabolic activity. Following port removal (Day 23) and multidisciplinary discussion, diagnosis of CRAT was retained. Antibiotics were stopped, and curative-dose apixaban was initiated (Day 28), with complete radiologic resolution at Day 27.

## Case presentation

**Figure 1:**
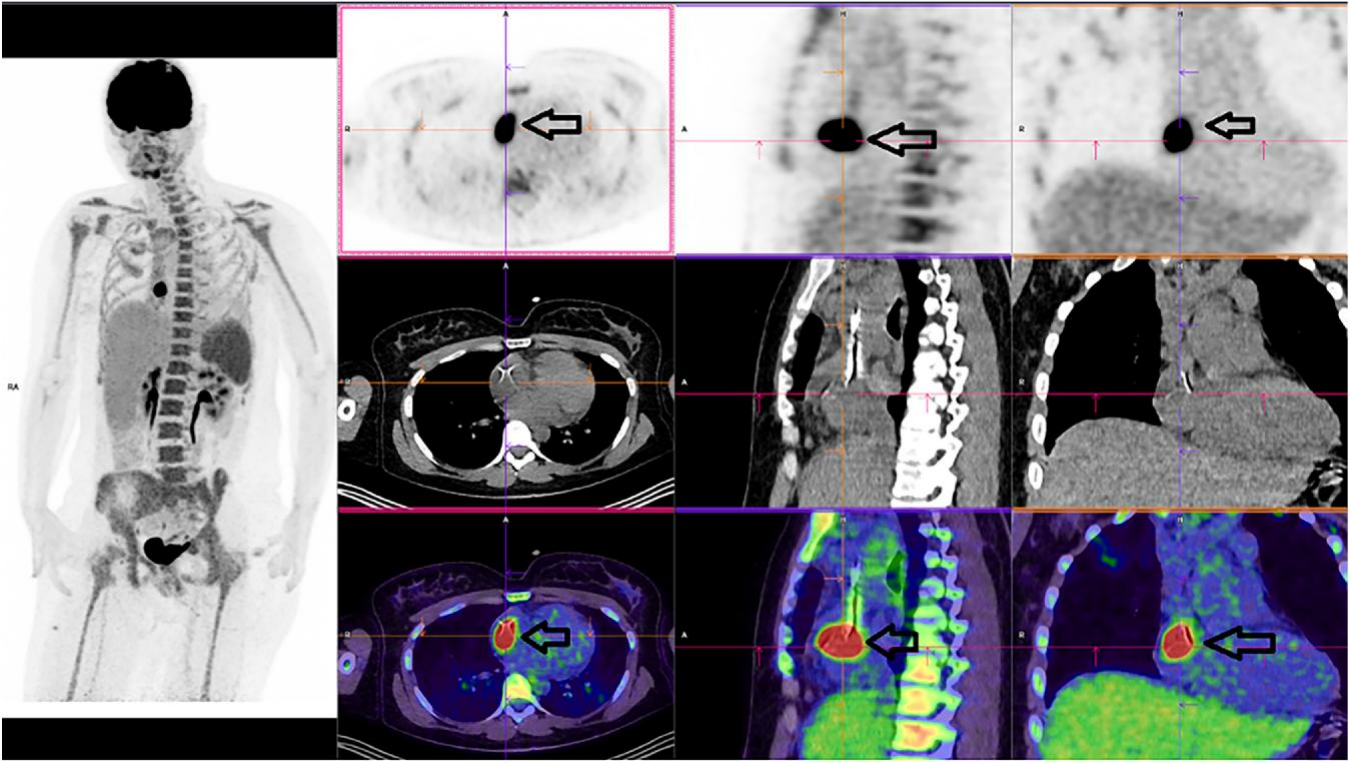
PET scan done at Day 7 showing images of hypermetabolism within the right atrium at the extremity of cath-a-port suggestive of infection of catheter thrombosis (see arrows).

A 29-year-old female undergoing BEACOPDac chemotherapy [B for bleomycin, E for etoposide, A for Adriamycin® (doxorubicin), C for cyclophosphamide, O for Oncovin® (vincristine), P for prednisolone, and D for dacarbazine] for Stage III Ab Hodgkin lymphoma, with recent reimplantation of a port-a-cath, presented with a 1-week history of fever and neutropenia. Initial blood cultures remained negative after starting broad-spectrum antibiotics (piperacillin–tazobactam). Positron emission tomography (PET) scan at day 7 suggested hypermetabolism at the catheter tip within the right atrium (*Figure 1*).

Physical examination at cardiology referral was unremarkable. Laboratory findings showed signs of inflammation: elevated C-reactive protein (normal range < 5 mg/L), 37 mg/L; white blood cell count (normal range 4.0–10.0 × 10^9^/L), 12.7 × 10^9^/L; and neutrophils (normal range 1.50–7.00 × 10^9^/L), 10.2 × 10^9^/L. Troponin T high-sensitivity was negative (normal range < 14 ng/L), 4 ng/L, and B-type natriuretic peptide was not elevated (normal range < 300 ng/L), 112 ng/L. Repeated daily blood cultures remained negative.

Electrocardiogram at admission shows sinus rhythm, normal axis, narrow QRS complex, no conduction disturbances, and no repolarization abnormalities.

**Figure 2:**
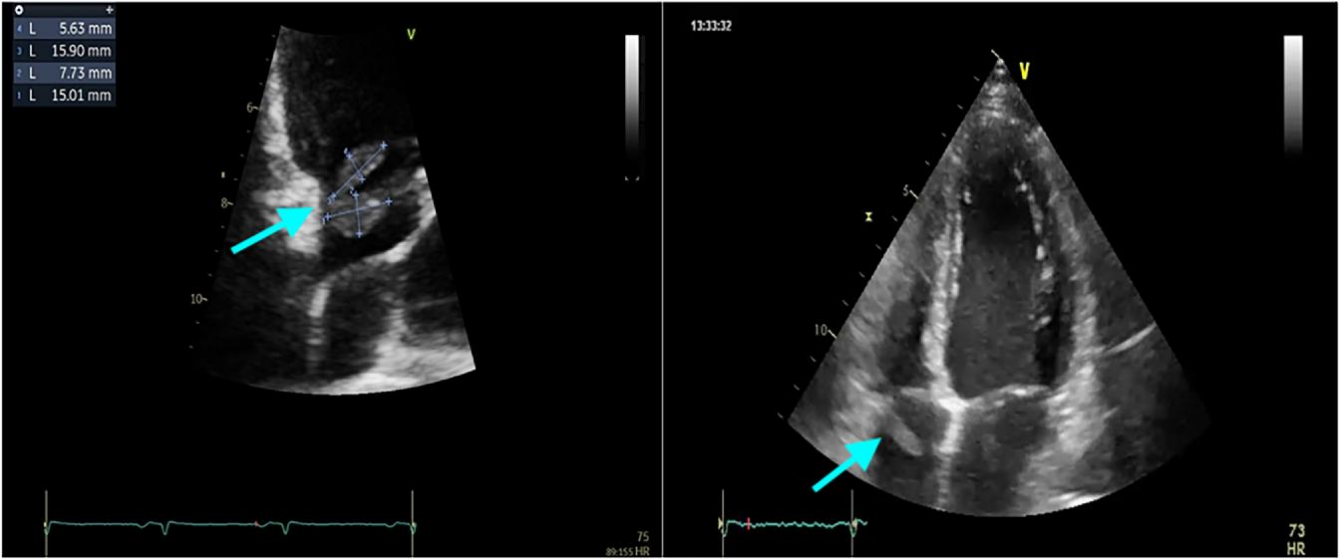
Images 1 and 2: transthoracic echocardiography at admission showing a mobile pediculated bilobed masse of diameter 9 × 14 mm and 6 × 14 mm attached to the lateral side of the tricuspid annulus within the right atrium in favour of vegetation and mild tricuspid regurgitation (see arrows).

Transthoracic echocardiography revealed a bilobed, pediculated mass (9 × 14 mm and 6 × 14 mm) attached to the lateral tricuspid annulus suggestive of vegetation, with mild tricuspid regurgitation. Left ventricle was non-dilated with preserved left ventricular ejection fraction 55%, absence of mitral or aortic abnormalities (*Figure 2*).

**Figure 3:**
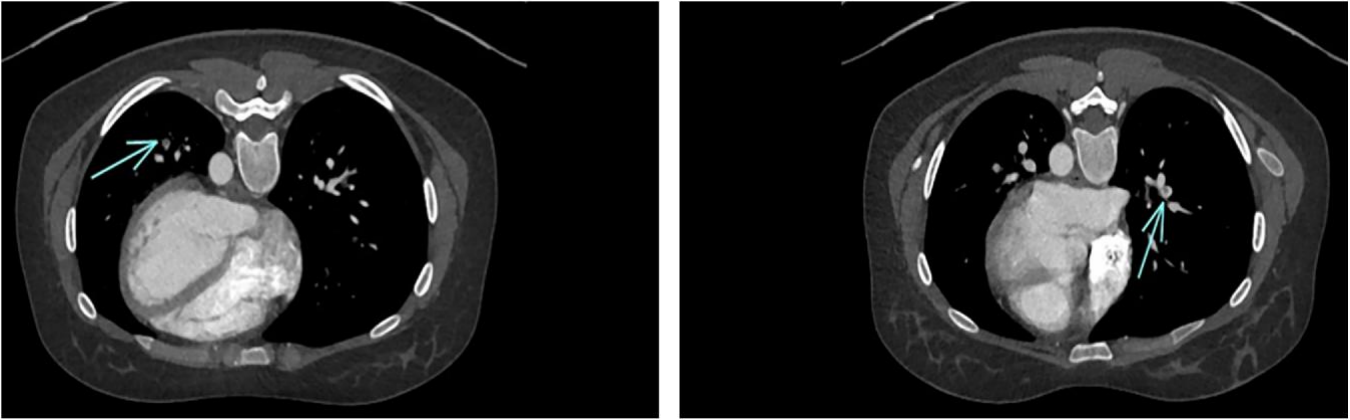
Thoraco-abdominopelvic CT scan at Day 20 revealed an endoluminal defect of the right inferior lobal artery, latero-basal segmentary, and left posture-basal artery, an image of webs seen at the right side, and absence of proximal pulmonary embolism (see arrows).

Computed tomography scan at Day 20 showed distal pulmonary embolism (*Figure 3*). The extremity of port-a-cath was seen at the junction of the SVC and right atrium without thrombus at the contact of catheter.

**Figure 4:**
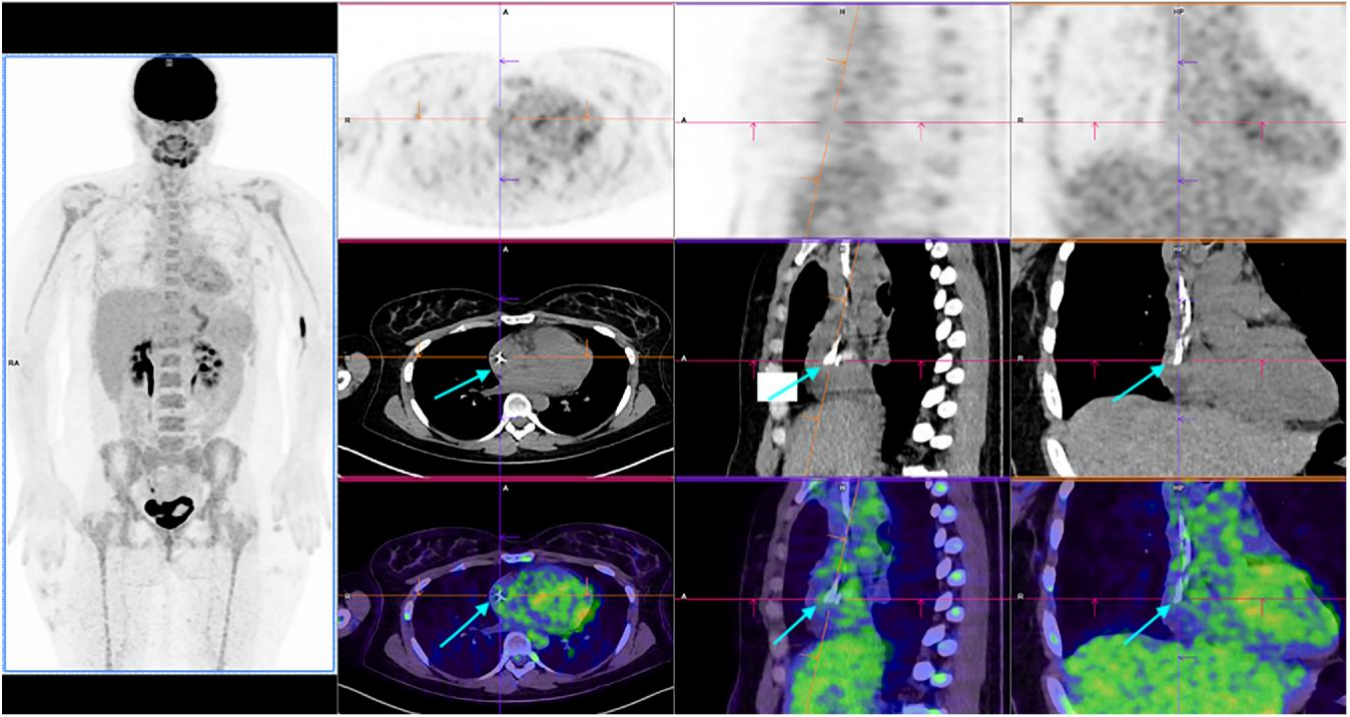
Follow-up PET scan at Day 20 showing complete absence of hypermetabolism at the catheter tip within the right atrium or other cardiac cavities (see arrows).

Follow-up PET scan at Day 20 demonstrated resolution of catheter hypermetabolism (*Figure 4*).

**Figure 5:**
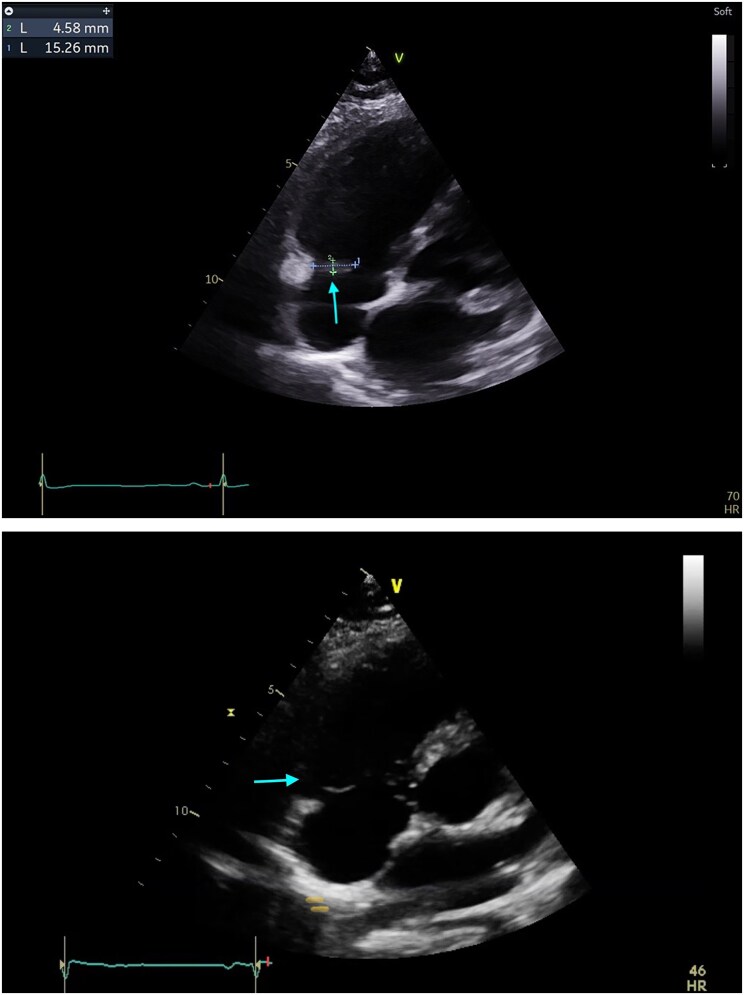
(*A*) Follow-up transthoracic echocardiography performed by the same operator after 1 week of anticoagulant at Day 21 on the Timeline revealed a persistence of one lobe (15 mm × 5 mm) pediculated mobile masse attached to the atrium side of the lateral tricuspid annulus (see arrows). One lobe of masse disappeared compared to the first transthoracic echocardiography (*Figure 2*). (*B*) Follow-up transthoracic echocardiography at Day 27 from initial presentation (18 March 2025), 4 days after removal of port-a-cath (14 March 2025), showed a complete resolution of the mass at the lateral side of the tricuspid annulus (see arrows).

Multidisciplinary discussion including oncology, cardiology, cardiac surgery, and infectious diseases suspended chemotherapy; initiated anticoagulation with low-molecular-weight heparin (LMWH) dose of 100 UI/kg/12 h (05 Mars 2025), at Day 14 on the Timeline; and escalated antibiotics to daptomycin, amoxicillin, and rifampicin. A follow-up TTE at Day 21 showed partial regression (one remaining pediculated mass of 15 × 5 mm) (*Figure 5A*).

The port-a-cath was removed at Day 23 and sent for culture, which also remained negative. A subsequent midline catheter for chemotherapy resumption also developed thrombosis.

At Day 27, TTE confirmed complete resolution of the intracardiac mass (*Figure 5B*). Final diagnosis was adjusted to CRAT with initial port-a-cath infection, effectively resolved following 28 days of antibiotic therapy, as evidenced by fever resolution, normalization of inflammatory biomarkers, and disappearance of hypermetabolism on subsequent PET imaging.

The patient was discharged at Day 28 with the resumption of chemotherapy, and enoxaparin was switched to a direct oral anticoagulant (DOAC) apixaban 5 mg b.i.d for a duration of 3 months. On 2 months of follow-up, the patient’s condition is favourable with a follow-up TTE at 2 months after discharge showing no abnormalities and pulmonary angiogram at 3 months showing absence of pulmonary embolism.

## Discussion

This case highlights the diagnostic dilemma between endocarditis and catheter-related thrombus on the tricuspid valve.

In the event of endocarditis, a 6-week antibiotic course would have been required, delaying essential lymphoma chemotherapy. A shorter 2-week regimen with close monitoring—though not supported by literature—could have been considered by analogy with some immunosuppressive protocols, possibly with growth factors to mitigate chemotherapy-related neutropenia.^3^

Conversely, in the case of a tricuspid thrombus, immediate anticoagulation and prompt chemotherapy were feasible, though with a risk of worsening possible undiagnosed endocarditis.

A combined initial strategy with antibiotics and anticoagulation was selected, with reassessment at 1 week. Rapid clinical improvement and mass resolution, confirmed by multidisciplinary discussion, supported CRAT. Antibiotics were stopped, and continued chemotherapy with favourable evolution confirmed the diagnosis.

For suspected IE, prolonged antibiotic therapy (typically 6 weeks) is recommended by the European Society of Cardiology (ESC) guidelines (2023).^4^ Delaying chemotherapy by 6 weeks, as per IE treatment guidelines, poses significant risks to patients with aggressive lymphomas, potentially adversely affecting prognosis according to the National Comprehensive Cancer Network (NCCN) guidelines (2023).^5^

The ESC guidelines (2023) recommend that surgical intervention in right-sided IE is typically considered if infection persists despite adequate antibiotic treatment, to prevent septic pulmonary embolism, or if severe tricuspid valve dysfunction occurs. Surgery is recommended for vegetations larger than 20 mm in case of multiple pulmonary embolism.^4^ In our case, these criteria were not met, negating surgical intervention. Current literature lacks explicit guidance regarding the safe timing to resume chemotherapy post-surgery for IE. A previous case involving a patient with treatment-resistant pemphigus showed safe reintroduction of immunosuppressive therapy 2 weeks post-vegetation removal surgery and 3 weeks after antibiotic initiation.^3^ However, the immunosuppressive intensity of BEACOPDac chemotherapy used for Hodgkin lymphoma significantly surpasses that of moderate-dose azathioprine and low-dose corticosteroids.^6,7^

Prompt removal of intravenous devices and subsequent cultures are essential in suspected right-sided IE, as guided by ESC 2015 recommendations, to facilitate targeted antibiotic therapy.^8^

Conversely, if intracardiac thrombus is suspected, immediate therapeutic anticoagulation is critical to prevent thromboembolic events, according to the 2019 ESC guidelines on thromboembolic disease.^9^ Routine replacement of intravenous catheters without confirmed infection remains controversial, but cultures from removed devices are crucial for infection exclusion.^10^

The observed mass was consistent with a catheter-related thrombus in the right atrium and tricuspid valve, likely induced by a ‘jet lesion’ mechanism, which is known to occur when the catheter tip is positioned directly within the right atrium, significantly increasing the risk of thrombus formation.^2^

Low-molecular-weight heparin remains preferred over DOAC in cancer patients, as per ASCO 2020 guidelines.^11^ Echocardiographic follow-up is key to assess thrombus evolution.

In our patient, thrombus size decreased after 1 week and resolved by 2 weeks of anticoagulation, allowing safe chemotherapy resumption. Absence of regression would have raised suspicion for IE.

A multidisciplinary team confirmed CRAT based on negative cultures, rapid thrombus resolution, and no signs of infection. Antibiotics were stopped, anticoagulation was continued, and monitoring ensured a favourable outcome at 2 months.

## Patient perspective

The patient felt reassured by the rapid diagnosis and the multidisciplinary approach that allowed timely chemotherapy resumption.

## Lead author biography


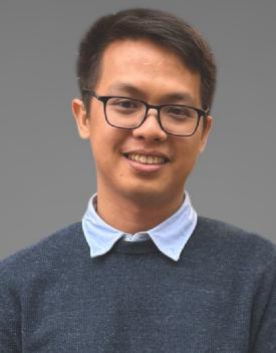


Sokpich Ren received his Bachelor Degree of Medicine from the University of Health Sciences and started his residency in cardiology in 2021 in Cambodia. Currently, he is training at the Cardiology Department of Kremlin-Bicêtre University Hospital, Assistance Publique-Hôpitaux de Paris (APHP).

## Supplementary Material

ytaf570_Supplementary_Data

## Data Availability

Additional data are available upon reasonable request.

## Notes

**Funding:** This report did not receive any specific funding.
